# Kernel canonical correlation analysis for assessing gene–gene interactions and application to ovarian cancer

**DOI:** 10.1038/ejhg.2013.69

**Published:** 2013-04-17

**Authors:** Nicholas B Larson, Gregory D Jenkins, Melissa C Larson, Robert A Vierkant, Thomas A Sellers, Catherine M Phelan, Joellen M Schildkraut, Rebecca Sutphen, Paul P D Pharoah, Simon A Gayther, Nicolas Wentzensen, Ovarian Cancer Association Consortium, Ellen L Goode, Brooke L Fridley

**Affiliations:** 1 Department of Health Sciences Research, Mayo Clinic, Rochester, MN, USA; 2 Cancer Epidemiology, Moffitt Cancer Center, Tampa, FL, USA; 3 Duke Comprehensive Cancer Center, Duke University, Durham, NC, USA; 4 Department of Pediatrics, Universty of South Florida College of Medicine, Tampa, FL, USA; 5 Department of Oncology, University of Cambridge, Cambridge, UK; 6 Department of Preventative Medicine, University of Southern California, Los Angeles, CA, USA; 7 Division of Cancer Epidemiology and Genetics, National Cancer Institute, Bethesda, MD, USA; 8 Department of Biostatistics, University of Kansas Medical Center, Kansas City, KS, USA

**Keywords:** association studies, canonical correlation, gene–gene interaction, kernel methods

## Abstract

Although single-locus approaches have been widely applied to identify disease-associated single-nucleotide polymorphisms (SNPs), complex diseases are thought to be the product of multiple interactions between loci. This has led to the recent development of statistical methods for detecting statistical interactions between two loci. Canonical correlation analysis (CCA) has previously been proposed to detect gene–gene coassociation. However, this approach is limited to detecting linear relations and can only be applied when the number of observations exceeds the number of SNPs in a gene. This limitation is particularly important for next-generation sequencing, which could yield a large number of novel variants on a limited number of subjects. To overcome these limitations, we propose an approach to detect gene–gene interactions on the basis of a kernelized version of CCA (KCCA). Our simulation studies showed that KCCA controls the Type-I error, and is more powerful than leading gene-based approaches under a disease model with negligible marginal effects. To demonstrate the utility of our approach, we also applied KCCA to assess interactions between 200 genes in the NF-κB pathway in relation to ovarian cancer risk in 3869 cases and 3276 controls. We identified 13 significant gene pairs relevant to ovarian cancer risk (local false discovery rate <0.05). Finally, we discuss the advantages of KCCA in gene–gene interaction analysis and its future role in genetic association studies.

## Introduction

Genome-wide association studies (GWAS) have identified hundreds of loci that harbor genetic variants that influence predisposition to a particular phenotype. Such studies involve the characterization of a large number of single-nucleotide polymorphisms (SNPs) across the genome and comparison of the frequency of variants across disease states. Initial GWAS analysis strategies involved single locus models, whereby individual markers were tested independently for association with a given phenotype. Although this approach has successfully identified regions of disease susceptibility, some contend it has failed to fully explain the heritability of complex phenotypes.^1, 2^ As common complex diseases and traits are thought to be a result of complex interactions and multiple low-penetrance variants,^3, 4^ multi-locus SNP models, as opposed to single SNP models, may better capture the true underlying genotypic–phenotypic relationship.

One strategy for multi-locus modeling is to jointly model the effects all SNPs within a given gene (eg, multivariable logistic regression models). However, this approach may lack power as the degrees of freedom of the model could be large and may require filtering or shrinkage approaches. Another drawback to the joint modeling of multiple SNPs within a gene is possible model fitting issues due to multicollinearity between SNPs (ie, linkage disequilibrium (LD)), as well as the lack of inclusion of LD information in the analysis. Recently, this idea of gene-level analysis has led to the concept of gene–gene interaction analysis, as opposed to SNP–SNP interaction approaches. Gene–gene interactions are not only hypothesized to have a large role in explaining missing heritability,^5^ they can also serve to provide biological information through construction of novel gene pathway topologies. Although classically gene–gene interactions have been defined statistically as deviance from additive marginal effects, such as in the case of logistic regression model, this type of model is limiting with respect to statistical power. Moreover, the results of such SNP–SNP interaction analyses lack clear biological interpretability.

Zhao *et al*^6^ proposed testing for interactions between two unlinked loci using measures of LD, which can be extended to case-control design by comparing such measures across case or disease status. This concept was adapted to gene-level analysis by Peng *et al*,^7^ which used a Wald-type *U*-statistic based on canonical correlation analysis^8^ (CCA) to detect gene–gene coassociation in case-control studies. As an LD-based procedure, the CCA approach obtains the maximal correlation of the linear combinations of the SNPs, coded as 0, 1 or 2 in terms of the minor allele, between two genes across case-control status, and tests whether the difference in the first canonical correlations is statistically significant. Although there are many benefits to this approach, it is limited to analyses where the number of observations exceeds the number of markers. Moreover, the use of CCA can only be used to detect linear relationships, which may limit power in the presence of nonlinear correlations between genes. Finally, CCA generally requires a large sample-to-feature ratio to avoid issues with overfitting the data, which raises questions of model regularization.

One solution to the above limitations involving the use of CCA for assessing gene–gene interactions is the use of kernels. Kernel methods are generally defined as algorithms that analyze data represented by similarity matrices, which are derived through the use of positive definite kernel functions.^9^ By mapping the original data to a nonlinear feature space, traditional linear methods involving dot products have been extended to nonlinear applications through the use of the ‘kernel trick'.^10^ The application of kernel machines is quite popular as a method to derive metrics of genomic similarity,^11^ and their use has been successful in the area of gene-level association analyses such as SKAT^12, 13^ and SPA-3G.^14^ Kernelized version of CCA (KCCA) provides a straightforward generalization of CCA to nonlinear correlations by applying CCA to kernel-generated feature spaces.

In this article, we develop a KCCA procedure for identifying coassociation between genes using genome-wide SNP data from a case-control study of complex phenotypes. We briefly discuss sample CCA and its kernelized version. We further outline and address the statistical and computational issues that accompany this approach, including concerns of regularization. To evaluate the properties of this method, we examine control of Type-I error rate and the power of our KCCA method compared with other existing methods for gene–gene interaction detection by a simulation study. Finally, we apply our KCCA approach to a study case-control study of invasive epithelial ovarian cancer to determine gene–gene interactions between genes within the NF-κB gene pathway.

## Materials and methods

### Data definition

Let 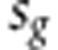 be the number of SNPs corresponding to the 
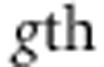
 gene in a given gene list of size *G*. Define 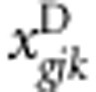 to be the genotype value for the 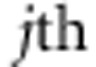 SNP in gene *g* in the 
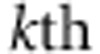
 case subject, for 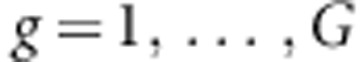, 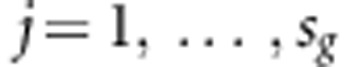, and 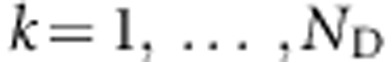, where 
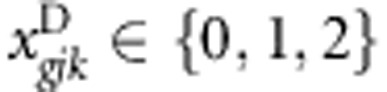
 is the number of copies of the minor allele for SNP *j* in gene *g* under an assumed joint additive-additive genetic model. Similarly, we define 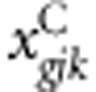 for the control subjects, where 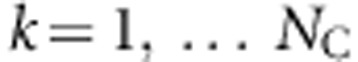. This genotypic information can in turn be represented by the respective 
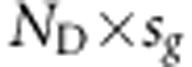
 and 
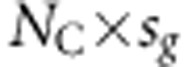
 matrices 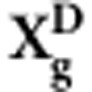 and 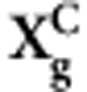. These matrices may also contain adjusted genotype values that have been corrected for various covariates and population stratification.

### Hypothesis testing

To test whether there is a statistical interaction between two genes across case-control status, we use KCCA to generate measures of genetic coassociation for both case and control status. For given genes *l*,*m*∈{1,...,*G*}, such that *l*≠*m*, consider the genotype matrices 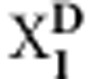, 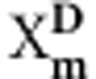, 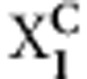, and 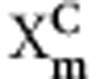, with corresponding reduced kernel representations 
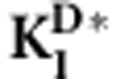
, 
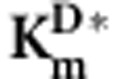
, 
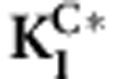
 , and 
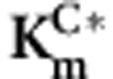
. Define 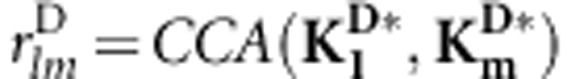 and 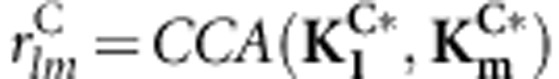 to be the respective maximal kernel canonical correlations for cases and controls between genes *l* and *m* (see Appendices I–III in Supplementary Material for details). We then define a statistic based upon an analog of the Fisher variance stabilizing transformation of the Pearson's correlation coefficient,^15^ given as


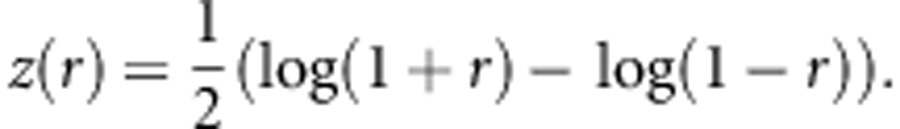


The transformation of the canonical correlation 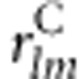 is written as 
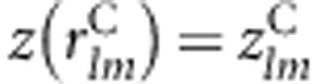
, which is approximately distributed as standard normal. A Wald-type statistic for assessing the statistical significance of the difference in gene–gene coassociation between cases and controls for genes *l* and *m* is defined by Peng *et al*^7^ as


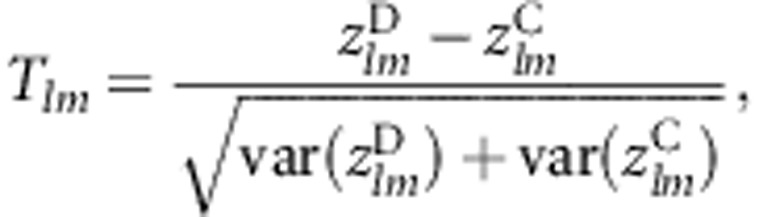


which is asymptotically distributed as *N*(0, 1) under the null hypothesis that 
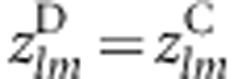
, and the cases and controls are independent. For estimating the transform variances 
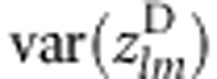
 and 
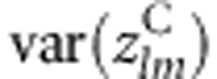
, we apply a robust resampling procedure, the trimmed jackknife^16^ (Appendix IV in Supplementary Materials).

### Multiple testing

For applications involving exhaustive hypothesis testing of all pairwise gene comparisons in a given gene list, multiple testing and test statistic correlation become problematic issues. To address both of these directly, we apply Efron's empirical null method^17^ for estimating the local false discovery rate (IFDR) for each hypothesis test conducted.

### Simulation study

To assess the properties of our KCCA procedure for gene–gene interaction testing, we consider simulation studies that evaluate type-I error control and power. We generated a population of haplotypes for two genes using real genotype data from our case study. fastPHASE^18^ was applied to the genotypes from the controls to estimate haplotypes for two genes of comparable size (25.9 and 30.6 kb), followed by use of HapSim^19^ to simulate 10 000 haplotypes for each gene. The respective numbers of polymorphic sites for each gene were 79 and 92. Genotype data for a hypothetical individual were simulated by combining pairs of randomly selected haplotypes for each gene.

Let 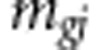 represent the derived minor allele frequency (MAF) from our simulated haplotype populations for marker *j* in gene *i*. Next, we randomly selected a fixed number of common (
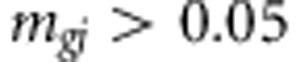
) markers to be causal for each gene. We then used a similar approach to effect size definition used by Wu *et al*,^12^ in which effects are a function of the MAF. Let 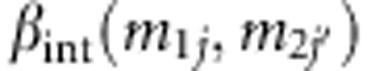 for an interaction effect, such that 
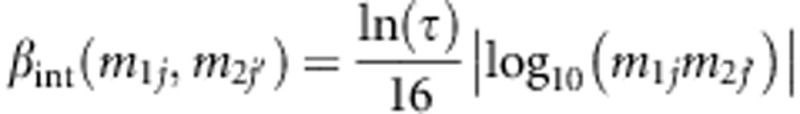
. Here 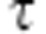 defines the maximum possible interaction effect. For example, for two markers with MAF=0.20 and *τ*=5, the interaction effect is 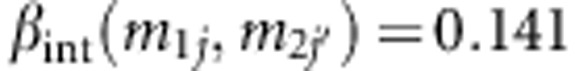.

Given the difficulty in genome-wide detection of gene–gene interactions without the presence of marginal effects, we only considered disease models with solely epistatic effects. We defined the probability of being a case conditional on genotype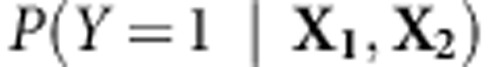 via a logistic regression framework, such that


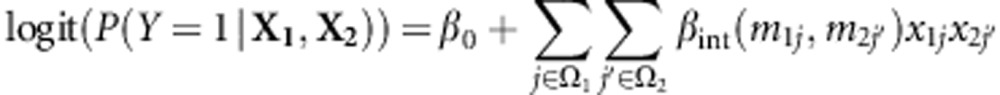


where Ω_1_ and Ω_2_ define the subsets of markers which are causal, and 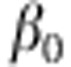 represents the disease prevalence. Sampling of cases and controls was then completed from a sufficiently large number of simulated genotype-phenotype pairs.

To comparatively evaluate the performance of our KCCA method, we included additional methods on the basis of similar analysis principles: the original CCA-based approach, PC-based logistic regression^20^ (PC-LR), and a composite-LD method^21^ (CLD). PC-LR obtains principal components from SNP measurements for each gene, which are then fit in simple logistic regression. The CLD method is a covariance-based approach, which evaluates the difference between block interactions across case-control status. Additional details for each approach can be found in their respective publications. For PC-LR, we evaluate the significance of the interaction coefficient between the first principal component of each gene, and for the CLD approach we use 5000 permutations to characterize the reference distribution of the test statistic. All declarations of statistical significance are made at an α-level of 0.05. For both Type-I error and power simulations, we consider whether or not explicit marginal effects are included in the disease model. Each simulation scenario is conducted with case-controls status sample sizes of 500, 1000, and 1500, with a total of 1000 iterations each.

### Ovarian cancer study

We applied the KCCA approach to detect gene–gene interaction within the NF-κB pathway, using data from a case-control study of invasive epithelial ovarian cancer as part of the GAME-ON Follow-up Ovarian Cancer Genetic Association and Interaction Studies collaboration (described elsewhere^22, 23^). Participants were enrolled in the Mayo Clinic Ovarian Cancer Study, North Carolina Ovarian Cancer Study, the Tampa Bay Ovarian Cancer Study, the Toronto Ovarian Cancer Study, the National Cancer Institute Ovarian Case-Control Study in Poland, the UK Ovarian Cancer Population Study, the Studies of Epidemiology and Risk Factors in Cancer Heredity Ovarian Cancer Study, the Familial Ovarian Cancer Registry Study, and the Royal Marsden Hospital Ovarian Cancer Study.^24, 25^ Study protocols were approved by the appropriate institutional review board or ethics panel, and all patients provided written informed consent. Genotypes were from the Illumina (San Diego, CA, USA) 610-Quad SNP arrays, with imputation to HapMap v 26 using MACH.^26^ For 200 autosomal genes within the NF-κB pathway, this resulted in ∼13 000 observed or imputed markers available on 3869 cases and 3276 controls of European descent. For genotyped markers, we coded genotypes as 0, 1, or 2 in terms of the number of observed minor alleles; for imputed markers, we used the expected genotype or ‘dosage'. Marker assignment to genes was determined on the basis of NCBI-build 36 gene location data, using a 20-kb buffer region on both the 5′ and 3′ ends of the defined gene location. Information on location, size, and number of SNPs in each of the 200 autosomal genes can be found in Supplementary Table S1.

To address the effects of possible confounding variables, we adjusted the genotypes for age, study site, and the first five principal components from an eigen analysis.^27^ Each unique gene pair was tested using the KCCA procedure, resulting in 
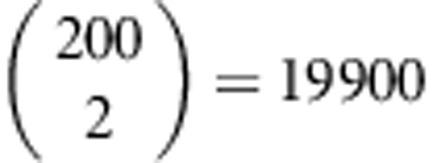
 total hypothesis tests. For purposes of comparison, we also applied the CCA-based procedure defined by Peng *et al*^7^ to the data, using 1000 bootstraps for variance estimation. Comparisons involving gene pairs with overlapping regions were removed from our analysis to avoid complications involving shared marker data.

## Results

### Type-I error

**Figure 1:**
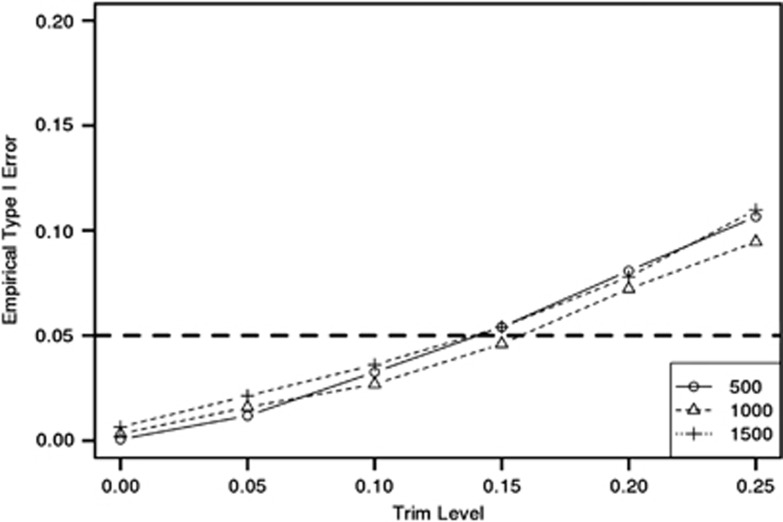
Line plot of the empirical Type-I error rates from the null simulations across trim levels *ω*=0.00, 0.05, 0.10, 0.15, 0.20, and 0.25, for sample sizes of 500, 1000, and 1500.

For our simulations, we considered six levels of trimming within the jackknife procedure for SE estimation to determine which level was appropriate. A plot of the empirical Type-I error rate for all trim levels across each sample size are found in Figure 1. The KCCA results derived from *ω*=0.15 yield near nominal Type-I error rate levels across all sample sizes. Detailed statistics on the empirical distributions of the test statistics can be found in Table 1. These results indicate that the test statistic follows the assumed standard normal distribution under the null.

### Power

**Figure 2:**
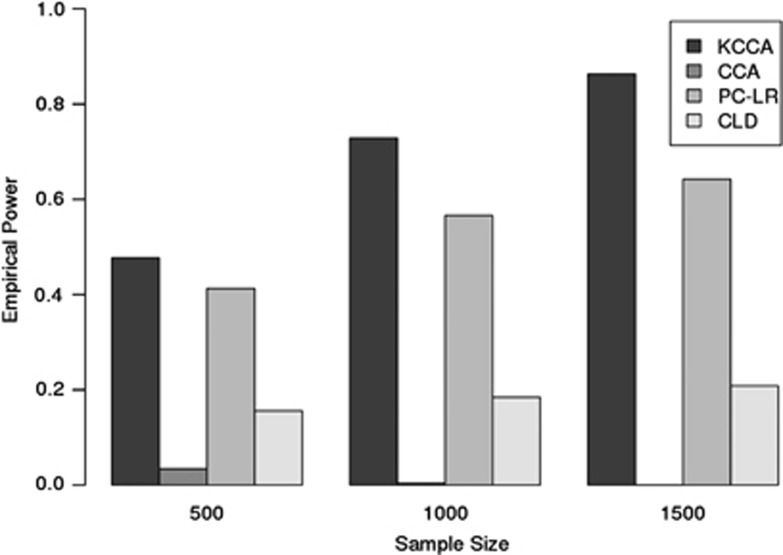
Barplots of the empirical power at *α*-level of 0.05 for the KCCA, CCA, PC-LR, and CLD gene–gene interaction methods, for sample sizes of 500, 1000, and 1500.

For our power simulations, we set 
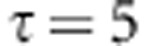
 and randomly selected five markers from each gene to be causal at each iteration, performing all KCCA tests with *ω*=0.15. Bar graphs of the results are found in Figure 2. From this plot, we observe that KCCA outperforms the other methods across all sample sizes, particularly the CCA approach. As the poor performance of CCA is likely due to issues with overfitting, we considered additional simulations where sample sizes were fixed at 1000 and the number of markers per gene was set to values of 10, 20, 30, 40, or 50, randomly subsetted from the total number of markers used in our simulations. Keeping all the other settings of our simulation design the same, the empirical power for each method across number of markers per gene is found in Table 2. Although the resulting power is much closer for low marker numbers, the KCCA approach is still consistently more powerful than the remaining methods.

### Application to ovarian cancer study

For the ovarian cancer study of the NF-κB pathway, we identified 13 statistically significant (lFDR<0.05) gene pairs of interest (Table 3) in applying our KCCA method, using the trimmed jackknife SE estimate with *ω*=0.15, with the top coassociated gene pair with case-control status occurring between *CASP8* and *MAP3K3*. Application of the CCA-based procedure resulted in 37 significant gene pairs; however, none of these overlapped with those detected by the KCCA method.

**Figure 3:**
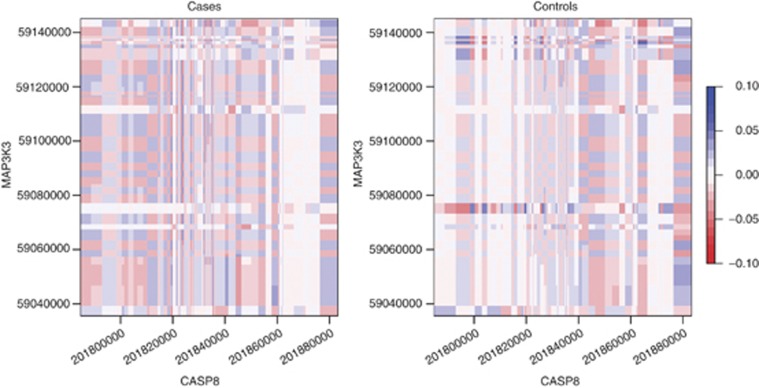
Colorized image plot of Pearson's correlation values between SNPs for *CASP8-MAP3K3* coassociation for cases (left) and controls (right). The axes depict the genomic position of the markers on the respective genes.

To explore one of the top coassociation hits at the SNP level, we analyzed the *CASP8*–*MAP3K3* interaction by generating pairwise marker Pearson's correlations. These are presented by case-control status in Figure 3 as gradient colorized images of the correlation coefficients. The figure demonstrates that although the Pearson's correlation coefficients themselves are small (|*ρ*|<0.10), there exist distinct differences between the two correlation structures. Of note is the discrepancy across case-control status with the correlation between marker *rs12940055* in *MAP3K3* (located at bp 59 075 874) and a large number of SNPs toward the 5′ end of *CASP8*, indicated by the horizontal band in the lower half of the correlation plots.

## Discussion

The identification of interactions between genes and their impact on complex diseases is adding to our understanding the genetic component. Although SNP-based interaction analysis methods are relatively well developed, the large number of SNPs in association studies makes exhaustive pairwise SNP-SNP analyses increasingly infeasible. By addressing the problem at the gene level, the scope of the analyses is not only computationally tenable, but also reduced to a biologically interpretable unit of interest.^28^ Peng *et al*^7^ presented a novel statistical method that allows for such analyses in their CCA statistic. However, as the number of genotyped markers increases, the application of CCA may be inappropriate, because the number of genotyped SNPs may approach or exceed the number of observations, especially for smaller experimental designs. This is of particular concern with post-GWAS whole exome and genome sequencing association studies, which will afford the characterization of additional variants unmeasured by current genotyping platforms.

In this article, we have presented a KCCA for gene–gene interaction analysis that not only addresses concerns of dimensionality, but also allows the flexibility to detect nonlinear correlations between genes. We have demonstrated that, using the appropriate SE estimation procedure, the KCCA test statistic exhibits near nominal levels of type-I error rate control and competitive power performance in our data simulations. We have also outlined basic procedures for large-scale application that take into account issues of regularization, computational burden, and multiple testing. As a result, the KCCA procedure is a powerful tool for exploratory gene–gene interaction analysis using SNP data.

**Figure 4:**
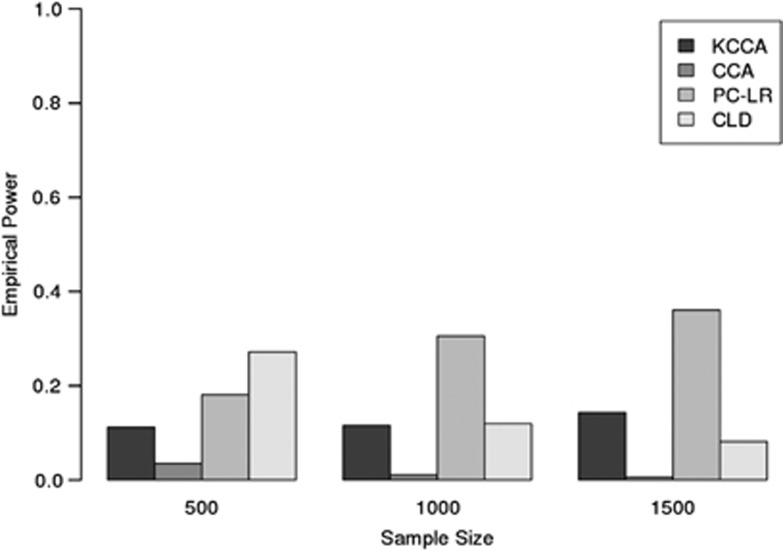
Barplots of the empirical power at *α*-level of 0.05 for the KCCA, CCA, PC-LR, and CLD gene–gene interaction methods, under sample sizes of 500, 1000, and 1500 with the inclusion of statistically significant marginal effects.

It is important to note that although we have shown via our simulation results that the performance of the gene-level interaction analyses using KCCA is more powerful than other current methods under an interaction-only model, additional simulations have shown that the PC-LR approach performs best in the presence of marginal effects (Figure 4). Thus, if evidence suggests the existence of such effects, we recommend the use of the PC-based procedure instead of KCCA. Also, if we collapse the signal into a single interaction between two markers with no LD present, individual SNP–SNP interaction analysis via logistic regression easily bests the gene-level analyses in interaction detection. Thus, although we have demonstrated that our KCCA method performs well as a genome-wide exploratory methodology, alternative methods may perform better under specific circumstances.

Although our data application analysis was conducted on genes within a specific pathway, we envision genome-wide exploratory applications, such as GWAS, to be completed in a similar fashion. However, owing to combinatorial scaling, this will require special computational considerations such as parallelization. For example, exhaustive pairwise analysis of a 20 000-gene genome requires nearly 200 million unique tests, although this total pales in comparison with the number of possible SNP–SNP interactions.

### Comparability with CCA statistic

Contrary to the findings of Peng *et al*,^7^ we have found that hypothesis testing using their CCA statistic with bootstrap variance estimation can be quite conservative, regardless of sample or feature size used, resulting in possible issues with reduced statistical power. This is evidenced in our power simulations where CCA power results for certain simulation conditions results in power below even nominal Type-I error rates. Upon investigation of this discrepancy, we found that the bootstrap variance estimates were quite large in comparison with their KCCA counterparts.

An additional benefit of our procedure is that the assumptions of CCA include multivariate normality of the observations, which is clearly violated by the discrete nature of genotype calls if no adjustments are made. Our method, however, involves low dimensional projections of kernelized observations, which has been shown to be approximately Gaussian.^29^ Thus, our KCCA procedure is also more consistent with the distributional assumptions of CCA.

### Ovarian cancer findings

The analysis of the FOCI data using the KCCA procedure yielded biologically interesting results, with many of the top gene pairs sharing some functional basis. *CASP8* and *MAP3K3* are integral members of the tumor necrosis factor pathway, and *IL1A* and *IL1B* both code for proinflammatory cytokines and have been jointly associated with lung cancer.^30^ With 13 statistically significant findings, there are also several novel interactions that may warrant further investigation.

We argue the lack of congruency between the results of the CCA-based procedure and our kernelized version is due, in large part, to the lack of any dimensional reduction in the CCA. Although the sample sizes used in our ovarian application analyses are large enough to satisfy most conventional notions of appropriate observation-to-feature ratios for accurate canonical correlation estimation, our simulations indicate that the CCA method suffers greatly from overfitting when there are a relatively large number of genotyped markers in given genes.

One significant gene pair of concern in the NF-κB–KCCA interaction analysis is *IL1A-IL1B*, which includes genes that are positionally adjacent to one another. Taking into account their respective buffer regions, only 4.4 kb separates the two genes. The significance of this gene pair could be evidence of instability of the method for gene pairs that are in high LD with each other because of locational proximity. However, gene pairs of this nature are also often closely linked functionally. Moreover, there are counter examples in our analysis of neighboring gene pairs that are statistically not significant (eg, *LTBR-TNFRSF1A*). Regardless, we recommend caution in the interpretation of such results.

### Conclusions and future development

Our KCCA algorithm simultaneously supplies dimensionality reduction and nonlinear coassociation analysis for high-dimensional SNP data, providing a powerful framework for detecting statistical epistasis at the gene level. Moreover, this type of analysis can isolate gene pairs of interest for follow-up analysis without being burdened by the multiple testing corrections necessary for genome-wide SNP–SNP pairwise interaction analysis. This is particularly relevant for next-generation sequencing applications, which may interrogate all possible SNPs through whole genome sequencing.

Although we have argued that the KCCA procedure for detecting gene–gene interaction possesses many advantages over the previously proposed CCA statistic, there is also room for improvement and generalizability of our approach. The use of the Gaussian kernel function is a robust selection; however, other kernel functions may be more appropriate for the specific data type, particularly if there are no adjustments for covariate data.^31^ The procedure itself may also be modified in a variety of ways, including the use of sparse canonical correlation^32, 33^ and multigene interaction analysis with generalized canonical correlation,^34^ and further exploring the resampling procedures used in the SE estimation. Finally, a less computationally intensive alternative to KCCA may be a kernelized variant of principal correlation,^35^ which could be considered for more demanding analyses such as genome-wide interrogation.

## Figures and Tables

**Table 1:** KCCA null simulation results for various trimming values ***ω***, which includes the *P*-value for the Kolmogorov-Smirnov test for normality (KS), the empirical SD and mean of the simulated test statistic distribution, as well as the realized Type-I error rate rejecting at an ***α***-level of 0.05

	*N*_C_*=N*_D_*=500*				*N*_C_*=N*_D_*=1000*				*N*_C_*=N*_D_*=1500*			
*ω*	*KS*	*SD*	*Mean*	*Type I*	*KS*	*SD*	*Mean*	*Type I*	*KS*	*SD*	*Mean*	*Type I*
0.00	<0.001	0.633	0.002	0.001	<0.001	0.643	0.001	0.003	<0.001	0.757	0.002	0.006
0.05	<0.001	0.798	−0.001	0.012	<0.001	0.801	−0.001	0.016	<0.001	0.938	−0.001	0.021
0.10	<0.001	0.904	−0.003	0.033	<0.001	0.902	−0.002	0.027	0.010	1.038	0.002	0.036
0.15	0.281	1.007	−0.003	0.540	0.810	1.008	−0.004	0.046	0.538	1.144	0.004	0.054
0.20	0.002	1.107	−0.001	0.081	0.002	1.114	−0.005	0.072	0.001	1.250	0.005	0.078
0.25	<0.001	1.201	−0.001	0.107	<0.001	1.220	−0.005	0.095	<0.001	1.363	0.004	0.110

**Table 2:** Simulation result for empirical power, using ***α*** =0.05 level significance testing, for the KCCA, CCA, PC-LR, and CLD testing procedures across varying numbers of markers per gene

	*Total markers per gene*				
	*10*	*20*	*30*	*40*	*50*
*KCCA*	0.852	0.818	0.799	0.779	0.757
*CCA*	0.428	0.097	0.012	0.002	0.000
*PC-LR*	0.570	0.575	0.552	0.545	0.544
*CLD*	0.724	0.570	0.371	0.305	0.234

Sample sizes are fixed at 1000, with five causal markers per gene.

**Table 3:** Detailed results of the significant KCCA gene–gene coassociations for analysis of ovarian cancer risk of significant (lFDR≤0.05) gene–gene interactions from FOCI analysis ranked by estimated lFDR

		*KCCA*					*CCA*	
*Gene 1*	*Gene 2*	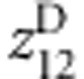	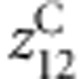	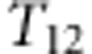	*P-value*	*lFDR*	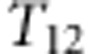	*P-value*
*CASP8*	*MAP3K3*	0.0875	0.0074	14.2897	<1.00E–16	3.38E–21	−0.7248	0.46853
*IL1A*	*IL1B*	1.7243	1.9167	−12.8110	<1.00E–16	1.58E–15	−0.0938	0.92525
*MAP3K3*	*TAB1*	0.0976	0.0196	8.1708	2.22E–16	8.53E–06	−0.2205	0.82548
*MAPK8*	*PELI3*	0.1146	0.0462	8.2271	2.22E–16	7.11E–06	−0.0947	0.92449
*AZI2*	*IKBKB*	0.1049	0.0344	9.2402	<1.00E–16	5.65E–08	0.2640	0.79171
*TBKBP1*	*TLR10*	0.0436	0.0989	−6.8641	6.69E–12	0.008161	−0.1386	0.88972
*HEXIM1*	*MYD88*	0.1073	0.0524	6.8256	8.75E–12	0.001646	−0.3888	0.69736
*PIK3CB*	*TAB1*	0.1025	0.0317	10.6548	<1.00E–16	2.59E–11	−0.2520	0.80098
*BIRC3*	*MAP3K3*	0.0763	0.0194	5.7655	8.14E–09	0.028977	0.7772	0.43702
*MAP3K1*	*TRAF7*	0.1125	0.0465	5.5000	3.80E–08	0.049916	−1.0316	0.30223
*BIRC3*	*TNFRSF13C*	0.0601	0.0001	10.8523	<1.00E–16	8.74E–12	−0.1986	0.84251
*MAP3K1*	*MAP3K8*	0.1032	0.0377	6.1801	6.41E–10	0.010925	0.3529	0.72412
*PYCARD*	*TAF4*	0.0231	0.1012	−6.5902	4.39E–11	0.016345	0.0313	0.97502

Abbreviations: CCA, canonical correlation analysis; FOCI, Follow-up Ovarian Cancer Genetic Association and Interaction Studies; KCCA, kernelized version of CCA; lFDR, local false discovery rate.

Includes the Fisher-transformed maximal kernel canonical correlation values for cases and controls, the resulting test statistic, *P*-values, and lFDR estimates for KCCA, as well as results for the CCA analysis.
