# Supplementary material for: Kernel canonical correlation analysis for assessing gene–gene interactions and application to ovarian cancer
Source: Eur J Hum Genet. 2013 Apr 17;22(1):126–31. doi: 10.1038/ejhg.2013.69 (PMC3865403; doi:10.1038/ejhg.2013.69)
Supplement: Supplementary Table S1 [file ejhg201369x1.doc]

**Supplementary Table**

**Table S1**: Details of 200 NF-κB genes included in ovarian risk analysis, including the number of genotyped markers mapped to each gene. The start and length data are based on the NCBI build 36 of the human genome.

| Gene | Chr. | Start bp | Size (kb) | Description | Markers |
| --- | --- | --- | --- | --- | --- |
| AHR | 7 | 17304832 | 47.467 | aryl hydrocarbon receptor | 40 |
| AKT1 | 14 | 104306731 | 26.394 | v-akt murine thymoma viral oncogene homolog 1 | 21 |
| AKT2 | 19 | 45428064 | 55.041 | v-akt murine thymoma viral oncogene homolog 2 | 34 |
| ARRB1 | 11 | 74654130 | 86.391 | arrestin, beta 1 | 105 |
| ARRB2 | 17 | 4560538 | 11.006 | arrestin, beta 2 | 33 |
| ATF1 | 12 | 49444086 | 57.088 | activating transcription factor 1 | 49 |
| ATM | 11 | 107598769 | 146.267 | ataxia telangiectasia mutated | 104 |
| ATR | 3 | 143650767 | 129.591 | ataxia telangiectasia and Rad3 related | 52 |
| AZI2 | 3 | 28339090 | 26.489 | 5-azacytidine induced 2 | 30 |
| BAG4 | 8 | 38153263 | 34.431 | BCL2-associated athanogene 4 | 20 |
| BCL10 | 1 | 85504047 | 12.124 | B-cell CLL/lymphoma 10 | 59 |
| BCL3 | 19 | 49943820 | 11.321 | B-cell CLL/lymphoma 3 | 13 |
| BIRC2 | 11 | 101723176 | 31.435 | baculoviral IAP repeat containing 2 | 36 |
| BIRC3 | 11 | 101693404 | 20.271 | baculoviral IAP repeat containing 3 | 18 |
| CAPN1 | 11 | 64705919 | 30.133 | calpain 1, (mu/I) large subunit | 48 |
| CARD10 | 22 | 36216346 | 28.81 | caspase recruitment domain family, member 10 | 65 |
| CARD11 | 7 | 2912295 | 137.81 | caspase recruitment domain family, member 11 | 228 |
| CARD14 | 17 | 75766876 | 30.57 | caspase recruitment domain family, member 14 | 63 |
| CARD6 | 5 | 40877167 | 14.046 | caspase recruitment domain family, member 6 | 44 |
| CARD8 | 19 | 53403325 | 41.412 | caspase recruitment domain family, member 8 | 91 |
| CARD9 | 9 | 138378229 | 9.71 | caspase recruitment domain family, member 9 | 51 |
| CASP1 | 11 | 104401445 | 9.622 | caspase 1, apoptosis-related cysteine peptidase (interleukin 1, beta, convertase) | 77 |
| CASP2 | 7 | 142695524 | 19.383 | caspase 2, apoptosis-related cysteine peptidase | 33 |
| CASP8 | 2 | 201806411 | 54.268 | caspase 8, apoptosis-related cysteine peptidase | 74 |
| CD247 | 1 | 165666505 | 87.945 | CD247 molecule | 195 |
| CD28 | 2 | 204279443 | 31.358 | CD28 molecule | 55 |
| CD3D | 11 | 117714999 | 3.67 | CD3d molecule, delta (CD3-TCR complex) | 25 |
| CD3E | 11 | 117680662 | 11.438 | CD3e molecule, epsilon (CD3-TCR complex) | 42 |
| CD3G | 11 | 117720311 | 8.95 | CD3g molecule, gamma (CD3-TCR complex) | 31 |
| CD40 | 20 | 44180313 | 11.478 | CD40 molecule, TNF receptor superfamily member 5 | 72 |
| CDC37 | 19 | 10362809 | 12.462 | cell division cycle 37 homolog (S. cerevisiae) | 15 |
| CFLAR | 2 | 201689135 | 48.125 | CASP8 and FADD-like apoptosis regulator | 35 |
| CHUK | 10 | 101938114 | 41.22 | conserved helix-loop-helix ubiquitous kinase | 70 |
| CRADD | 12 | 92595282 | 173.381 | CASP2 and RIPK1 domain containing adaptor with death domain | 208 |
| CSNK2A1 | 20 | 411338 | 61.144 | casein kinase 2, alpha 1 polypeptide | 74 |
| CSNK2A2 | 16 | 56749312 | 39.971 | casein kinase 2, alpha prime polypeptide | 71 |
| CSNK2B | 6 | 31741636 | 4.186 | casein kinase 2, beta polypeptide | 61 |
| CYLD | 16 | 49333462 | 59.885 | cylindromatosis (turban tumor syndrome) | 77 |
| ECSIT | 19 | 11477744 | 23.186 | ECSIT homolog (Drosophila) | 22 |
| EGF | 4 | 111053499 | 99.369 | epidermal growth factor | 151 |
| EGFR | 7 | 55054219 | 188.306 | epidermal growth factor receptor | 287 |
| EGR1 | 5 | 137829080 | 3.823 | early growth response 1 | 20 |
| EIF2AK2 | 2 | 37187203 | 50.369 | eukaryotic translation initiation factor 2-alpha kinase 2 | 63 |
| F2R | 5 | 76047542 | 19.512 | coagulation factor II (thrombin) receptor | 86 |
| FADD | 11 | 69726917 | 4.227 | Fas (TNFRSF6)-associated via death domain | 23 |
| FBXW7 | 4 | 153461860 | 213.762 | F-box and WD repeat domain containing 7 | 65 |
| G3BP2 | 4 | 76786990 | 30.639 | GTPase activating protein (SH3 domain) binding protein 2 | 30 |
| GSK3A | 19 | 47426178 | 12.398 | glycogen synthase kinase 3 alpha | 7 |
| GSK3B | 3 | 121028233 | 266.97 | glycogen synthase kinase 3 beta | 144 |
| HEXIM1 | 17 | 40580467 | 4.784 | hexamethylene bis-acetamide inducible 1 | 21 |
| HNRNPAB | 5 | 177564114 | 6.676 | heterogeneous nuclear ribonucleoprotein A/B | 63 |
| HRAS | 11 | 522242 | 3.308 | v-Ha-ras Harvey rat sarcoma viral oncogene homolog | 13 |
| IKBKAP | 9 | 110669621 | 66.596 | inhibitor of kappa light polypeptide gene enhancer in B-cells, kinase complex-associated protein | 208 |
| IKBKB | 8 | 42247986 | 61.136 | inhibitor of kappa light polypeptide gene enhancer in B-cells, kinase beta | 51 |
| IKBKE | 1 | 204710419 | 26.426 | inhibitor of kappa light polypeptide gene enhancer in B-cells, kinase epsilon | 88 |
| IL10 | 1 | 205007571 | 4.891 | interleukin 10 | 51 |
| IL12A | 3 | 161189323 | 7.177 | interleukin 12A (natural killer cell stimulatory factor 1, cytotoxic lymphocyte maturation factor 1, p35) | 81 |
| IL15 | 4 | 142777204 | 96.858 | interleukin 15 | 102 |
| IL17A | 6 | 52159144 | 4.251 | interleukin 17A | 54 |
| IL18 | 11 | 111519186 | 20.864 | interleukin 18 (interferon-gamma-inducing factor) | 51 |
| IL1A | 2 | 113247963 | 11.479 | interleukin 1, alpha | 56 |
| IL1B | 2 | 113303808 | 7.019 | interleukin 1, beta | 37 |
| IL1R1 | 2 | 102136834 | 25.932 | interleukin 1 receptor, type I | 105 |
| IL1R2 | 2 | 101974738 | 36.579 | interleukin 1 receptor, type II | 116 |
| IL1RN | 2 | 113591941 | 16.123 | interleukin 1 receptor antagonist | 95 |
| IL2 | 4 | 123592075 | 5.025 | interleukin 2 | 24 |
| IL3 | 5 | 131424246 | 2.549 | interleukin 3 (colony-stimulating factor, multiple) | 43 |
| IL6 | 7 | 22733343 | 4.802 | interleukin 6 (interferon, beta 2) | 80 |
| IL8 | 4 | 74825139 | 3.158 | interleukin 8 | 43 |
| ILK | 11 | 6581540 | 7.137 | integrin-linked kinase | 28 |
| IRAK2 | 3 | 10181563 | 78.864 | interleukin-1 receptor-associated kinase 2 | 83 |
| IRAK3 | 12 | 64869284 | 59.368 | interleukin-1 receptor-associated kinase 3 | 72 |
| IRAK4 | 12 | 42439047 | 29.119 | interleukin-1 receptor-associated kinase 4 | 81 |
| KPNA3 | 13 | 49171462 | 93.596 | karyopherin alpha 3 (importin alpha 4) | 100 |
| KRAS | 12 | 25249447 | 45.674 | v-Ki-ras2 Kirsten rat sarcoma viral oncogene homolog | 95 |
| LAT | 16 | 28903648 | 5.957 | linker for activation of T cells | 23 |
| LBP | 20 | 36408299 | 30.768 | lipopolysaccharide binding protein | 82 |
| LCK | 1 | 32489427 | 34.926 | lymphocyte-specific protein tyrosine kinase | 20 |
| LCP2 | 5 | 169607666 | 49.734 | lymphocyte cytosolic protein 2 (SH2 domain containing leukocyte protein of 76kDa) | 104 |
| LTA | 6 | 31648072 | 2.005 | lymphotoxin alpha (TNF superfamily, member 1) | 55 |
| LTB | 6 | 31656314 | 1.867 | lymphotoxin beta (TNF superfamily, member 3) | 53 |
| LTBR | 12 | 6363618 | 7.375 | lymphotoxin beta receptor (TNFR superfamily, member 3) | 26 |
| MALT1 | 18 | 54489598 | 78.752 | mucosa associated lymphoid tissue lymphoma translocation gene 1 | 76 |
| MAP2K1 | 15 | 64466679 | 104.257 | mitogen-activated protein kinase kinase 1 | 94 |
| MAP2K2 | 19 | 4041319 | 33.807 | mitogen-activated protein kinase kinase 2 | 45 |
| MAP2K3 | 17 | 21128561 | 30.584 | mitogen-activated protein kinase kinase 3 | 45 |
| MAP2K4 | 17 | 11864860 | 122.916 | mitogen-activated protein kinase kinase 4 | 92 |
| MAP2K6 | 17 | 64922433 | 127.632 | mitogen-activated protein kinase kinase 6 | 181 |
| MAP2K7 | 19 | 7874765 | 10.598 | mitogen-activated protein kinase kinase 7 | 17 |
| MAP3K1 | 5 | 56146657 | 81.079 | mitogen-activated protein kinase kinase kinase 1 | 122 |
| MAP3K11 | 11 | 65121802 | 16.494 | mitogen-activated protein kinase kinase kinase 11 | 23 |
| MAP3K14 | 17 | 40696271 | 53.926 | mitogen-activated protein kinase kinase kinase 14 | 48 |
| MAP3K2 | 2 | 127778609 | 38.631 | mitogen-activated protein kinase kinase kinase 2 | 34 |
| MAP3K3 | 17 | 59053533 | 73.869 | mitogen-activated protein kinase kinase kinase 3 | 44 |
| MAP3K7 | 6 | 91282074 | 71.554 | mitogen-activated protein kinase kinase kinase 7 | 178 |
| MAP3K8 | 10 | 30762872 | 27.895 | mitogen-activated protein kinase kinase kinase 8 | 58 |
| MAP4K1 | 19 | 43770121 | 30.362 | mitogen-activated protein kinase kinase kinase kinase 1 | 31 |
| MAPK1 | 22 | 20443946 | 108.024 | mitogen-activated protein kinase 1 | 100 |
| MAPK14 | 6 | 36103551 | 82.962 | mitogen-activated protein kinase 14 | 94 |
| MAPK3 | 16 | 30032927 | 9.204 | mitogen-activated protein kinase 3 | 10 |
| MAPK8 | 10 | 49279693 | 33.496 | mitogen-activated protein kinase 8 | 67 |
| MAVS | 20 | 3775484 | 20.489 | mitochondrial antiviral signaling protein | 35 |
| MDM2 | 12 | 67488247 | 32.234 | Mdm2 p53 binding protein homolog (mouse) | 37 |
| MTOR | 1 | 11089179 | 155.974 | mechanistic target of rapamycin (serine/threonine kinase) | 48 |
| MYD88 | 3 | 38155009 | 4.508 | myeloid differentiation primary response gene (88) | 14 |
| NCAM1 | 11 | 112337205 | 317.163 | neural cell adhesion molecule 1 | 488 |
| NFKB1 | 4 | 103641518 | 115.989 | nuclear factor of kappa light polypeptide gene enhancer in B-cells 1 | 179 |
| NFKB2 | 10 | 104144219 | 8.052 | nuclear factor of kappa light polypeptide gene enhancer in B-cells 2 (p49/p100) | 10 |
| NFKBIA | 14 | 34940468 | 3.227 | nuclear factor of kappa light polypeptide gene enhancer in B-cells inhibitor, alpha | 28 |
| NFKBIB | 19 | 44082455 | 8.919 | nuclear factor of kappa light polypeptide gene enhancer in B-cells inhibitor, beta | 33 |
| NFKBIE | 6 | 44333881 | 7.622 | nuclear factor of kappa light polypeptide gene enhancer in B-cells inhibitor, epsilon | 31 |
| NFKBIZ | 3 | 103029547 | 33.009 | nuclear factor of kappa light polypeptide gene enhancer in B-cells inhibitor, zeta | 39 |
| NKIRAS1 | 3 | 23908576 | 24.965 | NFKB inhibitor interacting Ras-like 1 | 42 |
| NKIRAS2 | 17 | 37422564 | 8.621 | NFKB inhibitor interacting Ras-like 2 | 14 |
| NOD1 | 7 | 30430675 | 54.115 | nucleotide-binding oligomerization domain containing 1 | 92 |
| NOD2 | 16 | 49288551 | 35.937 | nucleotide-binding oligomerization domain containing 2 | 58 |
| NR1D1 | 17 | 35502567 | 7.932 | nuclear receptor subfamily 1, group D, member 1 | 20 |
| OTUD7B | 1 | 148178855 | 70.455 | OTU domain containing 7B | 36 |
| PDPK1 | 16 | 2527971 | 65.219 | 3-phosphoinositide dependent protein kinase-1 | 3 |
| PELI1 | 2 | 64173499 | 19.488 | pellino homolog 1 (Drosophila) | 52 |
| PELI2 | 14 | 55654846 | 182.938 | pellino homolog 2 (Drosophila) | 324 |
| PELI3 | 11 | 65990912 | 10.472 | pellino homolog 3 (Drosophila) | 18 |
| PGR | 11 | 100414313 | 92.152 | progesterone receptor | 174 |
| PIK3CA | 3 | 180349005 | 86.189 | phosphoinositide-3-kinase, catalytic, alpha polypeptide | 65 |
| PIK3CB | 3 | 139856921 | 103.954 | phosphoinositide-3-kinase, catalytic, beta polypeptide | 58 |
| PIK3CG | 7 | 106293160 | 41.668 | phosphoinositide-3-kinase, catalytic, gamma polypeptide | 75 |
| PIK3R1 | 5 | 67558218 | 75.187 | phosphoinositide-3-kinase, regulatory subunit 1 (alpha) | 104 |
| PKN1 | 19 | 14405166 | 38.513 | protein kinase N1 | 41 |
| PLCG1 | 20 | 39199575 | 38.196 | phospholipase C, gamma 1 | 38 |
| PPARA | 22 | 44925163 | 93.154 | peroxisome proliferator-activated receptor alpha | 93 |
| PPARG | 3 | 12304349 | 146.506 | peroxisome proliferator-activated receptor gamma | 171 |
| PPP1R13L | 19 | 50574736 | 25.393 | protein phosphatase 1, regulatory (inhibitor) subunit 13 like | 31 |
| PRKACA | 19 | 14063500 | 26.059 | protein kinase, cAMP-dependent, catalytic, alpha | 10 |
| PRKACB | 1 | 84316333 | 160.436 | protein kinase, cAMP-dependent, catalytic, beta | 134 |
| PRKCA | 17 | 61729388 | 507.936 | protein kinase C, alpha | 622 |
| PRKCQ | 10 | 6509111 | 153.133 | protein kinase C, theta | 231 |
| PRKCZ | 1 | 1971769 | 134.925 | protein kinase C, zeta | 78 |
| PRKDC | 8 | 48848222 | 187.074 | protein kinase, DNA-activated, catalytic polypeptide | 52 |
| PTPN13 | 4 | 87734909 | 220.417 | protein tyrosine phosphatase, non-receptor type 13 (APO-1/CD95 (Fas)-associated phosphatase) | 147 |
| PYCARD | 16 | 31120308 | 1.444 | PYD and CARD domain containing | 6 |
| RAF1 | 3 | 12600108 | 80.57 | v-raf-1 murine leukemia viral oncogene homolog 1 | 96 |
| REL | 2 | 60962256 | 41.426 | v-rel reticuloendotheliosis viral oncogene homolog (avian) | 39 |
| RELA | 11 | 65178393 | 8.558 | v-rel reticuloendotheliosis viral oncogene homolog A (avian) | 15 |
| RELB | 19 | 50196552 | 36.74 | v-rel reticuloendotheliosis viral oncogene homolog B | 32 |
| RHOA | 3 | 49371582 | 52.948 | ras homolog gene family, member A | 38 |
| RIPK1 | 6 | 3022057 | 38.363 | receptor (TNFRSF)-interacting serine-threonine kinase 1 | 34 |
| RIPK2 | 8 | 90839110 | 33.323 | receptor-interacting serine-threonine kinase 2 | 67 |
| RIPK3 | 14 | 23875067 | 4.015 | receptor-interacting serine-threonine kinase 3 | 57 |
| RNF216 | 7 | 5626204 | 161.614 | ring finger protein 216 | 166 |
| RNF25 | 2 | 219236831 | 8.194 | ring finger protein 25 | 35 |
| RPS6KA1 | 1 | 26728836 | 45.271 | ribosomal protein S6 kinase, 90kDa, polypeptide 1 | 53 |
| RPS6KA4 | 11 | 63883201 | 13.062 | ribosomal protein S6 kinase, 90kDa, polypeptide 4 | 22 |
| RPS6KA5 | 14 | 90406912 | 189.834 | ribosomal protein S6 kinase, 90kDa, polypeptide 5 | 151 |
| SOD1 | 21 | 31953806 | 9.309 | superoxide dismutase 1, soluble | 21 |
| SPAG9 | 17 | 46397987 | 155.107 | sperm associated antigen 9 | 69 |
| STAT1 | 2 | 191542121 | 45.06 | signal transducer and activator of transcription 1, 91kDa | 81 |
| STAT3 | 17 | 37718869 | 75.17 | signal transducer and activator of transcription 3 (acute-phase response factor) | 50 |
| TAB1 | 22 | 38125705 | 37.373 | TGF-beta activated kinase 1/MAP3K7 binding protein 1 | 42 |
| TAB2 | 6 | 149680756 | 93.684 | TGF-beta activated kinase 1/MAP3K7 binding protein 2 | 165 |
| TAF3 | 10 | 7900679 | 196.041 | TAF3 RNA polymerase II, TATA box binding protein (TBP)-associated factor, 140kDa | 290 |
| TAF4 | 20 | 59983249 | 91.012 | TAF4 RNA polymerase II, TATA box binding protein (TBP)-associated factor, 135kDa | 96 |
| TANK | 2 | 161701712 | 99.216 | TRAF family member-associated NFKB activator | 88 |
| TBK1 | 12 | 63132204 | 49.954 | TANK-binding kinase 1 | 47 |
| TBKBP1 | 17 | 43127629 | 16.786 | TBK1 binding protein 1 | 28 |
| TDP2 | 6 | 24758184 | 16.91 | tyrosyl-DNA phosphodiesterase 2 | 59 |
| TICAM1 | 19 | 4766992 | 2.459 | toll-like receptor adaptor molecule 1 | 29 |
| TICAM2 | 5 | 114942247 | 47.363 | toll-like receptor adaptor molecule 2 | 60 |
| TIFA | 4 | 113416231 | 10.277 | TRAF-interacting protein with forkhead-associated domain | 42 |
| TIRAP | 11 | 125658192 | 11.846 | toll-interleukin 1 receptor (TIR) domain containing adaptor protein | 58 |
| TLR1 | 4 | 38474271 | 8.536 | toll-like receptor 1 | 61 |
| TLR10 | 4 | 38450629 | 10.355 | toll-like receptor 10 | 78 |
| TLR2 | 4 | 154824891 | 21.802 | toll-like receptor 2 | 56 |
| TLR3 | 4 | 187227303 | 15.943 | toll-like receptor 3 | 35 |
| TLR4 | 9 | 119506431 | 13.158 | toll-like receptor 4 | 68 |
| TLR5 | 1 | 221350207 | 33.04 | toll-like receptor 5 | 1 |
| TLR6 | 4 | 38504803 | 2.752 | toll-like receptor 6 | 31 |
| TLR9 | 3 | 52230138 | 5.081 | toll-like receptor 9 | 23 |
| TNF | 6 | 31651329 | 2.762 | tumor necrosis factor | 55 |
| TNFAIP3 | 6 | 138230274 | 15.868 | tumor necrosis factor, alpha-induced protein 3 | 34 |
| TNFRSF10A | 8 | 23104915 | 33.669 | tumor necrosis factor receptor superfamily, member 10a | 55 |
| TNFRSF10B | 8 | 22933591 | 49.046 | tumor necrosis factor receptor superfamily, member 10b | 49 |
| TNFRSF10D | 8 | 23049046 | 28.439 | tumor necrosis factor receptor superfamily, member 10d, decoy with truncated death domain | 27 |
| TNFRSF11A | 18 | 58143528 | 60.956 | tumor necrosis factor receptor superfamily, member 11a, NFKB activator | 160 |
| TNFRSF13B | 17 | 16783123 | 33.004 | tumor necrosis factor receptor superfamily, member 13B | 82 |
| TNFRSF13C | 22 | 40650982 | 1.746 | tumor necrosis factor receptor superfamily, member 13C | 16 |
| TNFRSF1A | 12 | 6308184 | 13.338 | tumor necrosis factor receptor superfamily, member 1A | 37 |
| TNFRSF1B | 1 | 12149647 | 42.217 | tumor necrosis factor receptor superfamily, member 1B | 67 |
| TNFSF10 | 3 | 173706158 | 17.805 | tumor necrosis factor (ligand) superfamily, member 10 | 99 |
| TNFSF11 | 13 | 42043795 | 36.353 | tumor necrosis factor (ligand) superfamily, member 11 | 93 |
| TOLLIP | 11 | 1252177 | 35.238 | toll interacting protein | 25 |
| TRADD | 16 | 65745589 | 5.724 | TNFRSF1A-associated via death domain | 16 |
| TRAF1 | 9 | 122704493 | 24.501 | TNF receptor-associated factor 1 | 64 |
| TRAF2 | 9 | 138900786 | 40.102 | TNF receptor-associated factor 2 | 39 |
| TRAF3 | 14 | 102313569 | 128.812 | TNF receptor-associated factor 3 | 92 |
| TRAF3IP2 | 6 | 111986836 | 47.178 | TRAF3 interacting protein 2 | 86 |
| TRAF5 | 1 | 209566580 | 48.331 | TNF receptor-associated factor 5 | 56 |
| TRAF6 | 11 | 36467302 | 21.096 | TNF receptor-associated factor 6 | 38 |
| TRAF7 | 16 | 2145800 | 22.331 | TNF receptor-associated factor 7 | 19 |
| TRIP6 | 7 | 100302886 | 6.126 | thyroid hormone receptor interactor 6 | 29 |
| ZAP70 | 2 | 97696463 | 26.292 | zeta-chain (TCR) associated protein kinase 70kDa | 45 |
